# A Novel Operation

**Published:** 1898-04

**Authors:** 


### A NOVEL OPERATION. REMOVAL OF BOTH SEMINAL VESICLES THROUGH THE BACK.

This operation was performed, so far as we know for the first time, on March 23, 1898, by Dr. Robert T. Morris, at the private Park Hospital, 132 East 16th Street, New York. The patient was 24 years of age. The vesicles were closed from gonorrhœal infection of long standing, and incapacitated the patient entirely from any active duty. The chief indication for operation was bowel obstruction due to a pelvic cellulitis, with pelvic peritonitis, very similar indeed to the symptoms of pyosalpinx in the female. Incision was made through the gluteus maximus exterior to the coccyx, above the anus, but avoiding the cutting of the fibres of the internal sphincter, and coccyx was removed with scissors: the dissection continued until the seminal vesicles were found firmly adherent to the surrounding structures. With great difficulty the adhesions were broken off, the vesicles freed and removed by enucleation. The specimens were about four times their normal size and much disorganized from inflammation. The operation is novel, was designed by Dr. Morris, and great skill was displayed in its execution. The patient is making a good recovery.

